# Pre-sleep heart rate variability predicts chronic insomnia and measures of sleep continuity in national-level athletes

**DOI:** 10.3389/fphys.2025.1627287

**Published:** 2025-09-18

**Authors:** Qinlong Li, Xiaochen Lei, Wenlang Yu, Charles J. Steward, Yue Zhou

**Affiliations:** ^1^ Sport Science School, Beijing Sport University, Beijing, China; ^2^ Sports and Health Laboratory, Beijing Xiaomi Mobile Software Co., Ltd., Beijing, China; ^3^ School of Life Sciences, University of Nottingham, Nottingham, United Kingdom

**Keywords:** chronic insomnia, athlete, heart rate variability, autonomic nervous system, linear regression model

## Abstract

**Objective:**

This study aimed to investigate whether pre-sleep heart rate variability (HRV) could predict chronic insomnia (CI) and sleep quality in male national-level team-based athletes.

**Methods:**

A total of 174 athletes participated in this study, including 98 with CI and 76 exhibiting normal sleeping patterns. Pre-sleep HRV was assessed using heart rate chest straps, and sleep quality was evaluated through polysomnography (PSG) before a single night’s sleep. Binary logistic regression was first used to predict CI. Multiple linear regression and multi-layer perceptron (MLP) neural network models were then used to predict measures of sleep quality.

**Results:**

Binary logistic regression revealed that measures of pre-sleep HRV accurately predict CI (*R* ^2^ = 0.902 and 96% accuracy, AUC = 0.997). Multiple linear regression showed that pre-sleep HRV had a moderate predictive capacity for time awake (*R* ^2^ = 0.526, *P* < 0.001) and sleep efficiency (*R* ^2^ = 0.481, *P* < 0.001). The multiple linear regression model’s predicted values for sleep onset latency (r = 0.459, *P* < 0.01), sleep efficiency (r = 0.554, *P* < 0.001), and deep sleep time (r = 0.536, *P* < 0.001) showed moderate positive correlations with the corresponding actual values, whereas the MLP neural network’s predictions were not significantly correlated with the actual values. In contrast, the MLP neural network model was superior at predicting time awake when compared to the multiple linear regression model (MLP: mean absolute percentage error = 0.182 vs. Multiple linear regression: mean absolute percentage error = 0.516).

**Conclusion:**

The present findings support the use of pre-sleep HRV not only to predict CI, but also some sleep continuity measures in national level athletes.

## 1 Introduction

Chronic insomnia (CI) is the most common sleep disorder, affecting approximately 10% of the population (Morin and Jarrin, 2022). It is characterized by persistent difficulty falling or staying asleep at least three times per week, for a period of 3 months or longer (Perlis et al., 2005). Elite athletes have a higher prevalence of sleep disorders than the general population due to overtraining, inadequate recovery, travel and performance pressure (Walsh et al., 2021). It is estimated that 13%–70% of athletes experience some form of sleep disruption, with 22%–26% suffering from severe sleep issues (Gupta et al., 2017). This is highly problematic given that poor sleep impairs athletic performance by reducing endurance, strength, speed, and reaction time, while increasing perceived effort and the risk of injury (Charest and Grandner, 2022). It also disrupts cognitive function, such as attention and decision-making, whilst also slowing recovery by altering hormonal balance and immune responses (Walsh et al., 2011). Therefore, finding strategies that assist with the early detection of CI and poor sleep quality remains a key focus for coaches, practitioners and sport scientists.

Commonly used tools for detecting sleep disturbances in athletes include the Pittsburgh Sleep Quality Index (PSQI) and actigraphy (Walsh et al., 2021). While these methods are practical and offer useful insights into sleep quality, polysomnography (PSG) remains the “gold standard” for the accurate diagnosis of sleep disorders as it provides more precise measurements of sleep architecture and disturbances through directly recording brain activity, eye movements, and muscle tone during sleep (Sateia, 2014). However, solely relying on sleep monitoring may delay the early diagnosis and treatment of long-term sleep conditions, such as CI (Garbarino and Bragazzi, 2024). Therefore, establishing prediction models for CI based on PSG data is essential, as they may offer a promising tool for the early diagnosis of CI. This would enable the personalized timing of CI treatment for individual athletes before significant declines in athletic performance occur.

Heart rate variability serves as a useful measure of autonomic nervous system function (Lehrer and Gevirtz, 2014), particularly in understanding sleep-related neural regulation through the balance between the sympathetic and parasympathetic branches (Li et al., 2024). Enhanced parasympathetic activity, reflected by higher high-frequency components of HRV, has been associated with the onset of sleep and a higher proportion of deep sleep (Grimaldi et al., 2019). In contrast, increased sympathetic activity is linked to difficulties in falling asleep and a higher proportion of light sleep (Michael et al., 2017). Long-term exercise training at appropriate frequencies, intensities, and volumes can lead to adaptive changes in HRV, whereas overtraining has been shown to have maladaptive effects on HRV (Marzbani et al., 2016). Notably, pre-sleep HRV biofeedback or relaxation techniques, such as controlled breathing and meditation, have been found to improve sleep quality by modulating HRV (Marzbani et al., 2016). These findings suggest that HRV may not only influence sleep regulation but could also have a role as a physiological predictor of sleep quality and CI.

This study aimed to investigate whether pre-sleep HRV could predict CI and various indicators of sleep quality in male national level team-sport athletes. Specifically, we sought to determine whether pre-sleep HRV is associated with the presence of CI and with key indicators of sleep quality. Additionally, we aimed to compare the predictive effectiveness of different modeling approaches. It was hypothesized that measures of pre-sleep HRV would serve as reliable predictors of CI and sleep quality outcomes.

## 2 Methods

### 2.1 Ethical approval

The study was approved by the Ethics Committee of the Beijing Sport University Sports Science Laboratory (Ethics Approval Number: 2023277H). All participants were fully informed of the experimental requirements and procedures and provided written informed consent prior to participation. This study was carried out in accordance with the principles outlined in the Declaration of Helsinki, except for prior registration in a database.

### 2.2 Participants

The sample size for this study was determined based on a multiple linear regression approach with a fixed model (G*Power 3.1), with 12 predictors *R* ^2^ representing the deviation from 0. Calculations were based on correlations between sleep onset latency (SOL), time awake (TA), and pre-sleep HRV (Li et al., 2022). The study was powered *a priori* to detect effect sizes of 0.3, with α = 0.05 and β = 0.95. This resulted in a minimum sample size of 23 for SOL and 82 for TA. To account for attrition, recruitment was increased by 20%, leading to a required minimum sample size of 102.

Only male national-level athletes (Tier 3) (McKay et al., 2022), between the ages of 18–25 years, who participated in team sports were recruited for the study. The inclusion criteria for CI athletes were based on the International Classification of Sleep Disorders-Third Edition (ICSD-3) (Sateia, 2014) and a Pittsburgh Sleep Quality Index (PSQI) score of >7. In addition, athletes were required to have no recent history of psychological stress (e.g., family accidents, unemployment, major social conflicts), and potential sleep disturbances caused by emotional disorders. Anxiety and depression were assessed through the Hamilton Anxiety Rating Scale (HAMA) and Hamilton Depression Rating Scale (HAMD) with scores <7 required to enroll onto the study. Participants were not taking psychiatric medications (e.g., sleeping medication), did not habitually nap in the day, and were regularly training.

In total, 174 male national-level athletes were enrolled (age: 20 ± 1 year; body mass: 76 ± 9 kg; height: 183 ± 8 cm; training: 4–5 times/week; sport-specific training duration: 470 ± 30 min/week) onto the study. The types of team sports included basketball, soccer, volleyball, rugby and ice hockey. Data collection was conducted between November 2023 and November 2024 as this was off season and therefore avoided long-distance travel, which would cause jet lag or circadian rhythm disruption, and irregular training schedules.

### 2.3 Experimental protocol

Participants completed inclusion and exclusion screening, informed consent forms, and were briefed on experimental procedures. To avoid non-standard sleep patterns, participants slept 1–2 nights in the sleep laboratory with monitoring equipment prior to commencing the study (Hu et al., 2024). To ensure participants were not experiencing sleep disturbances prior to the study, which included two laboratory familiarization nights, we compared PSQI scores from the post-familiarization period to those at enrollment, with each score reflecting the preceding 1-month period. On testing days, athletes avoided intense physical activity for 24 h. Athletes were also advised to refrain from consuming foods and beverages known to disrupt sleep, such as caffeine and spicy foods and to limit excessive screen time by avoiding the use of devices such as mobile phones and laptops over the 4 h before bedtime. Participants arrived at the sleep laboratory 45–60 min before their usual bedtime. Prior to sleep, anthropometric data were collected and HRV was measured before the PSG was attached for overnight sleep monitoring. During sleep monitoring, participants were not restricted to specific sleep or wake times and were instructed to fall asleep and wake up naturally.

### 2.4 Sleep quality monitoring and evaluation

#### 2.4.1 Subjective sleep quality

The PSQI was used to assess sleep quality (BUYSSE and REYNOLDS, 1989) and included seven components: subjective sleep quality, sleep latency, sleep duration, sleep efficiency, sleep disturbances, use of sleeping medication, and daytime dysfunction. Each component is scored on a scale from 0 to three and the total PSQI score was calculated by summing the scores of all components. As such, scores can range from 0 to 21, with higher scores indicating greater sleep impairment (Mollayeva et al., 2016).

#### 2.4.2 Objective sleep quality

A PSG system (Natus Medical Inc., San Carlos, Canada) recorded sleep using electroencephalogram (EEG) (C4), electrooculogram (EOG) (below the left and above the right eye), and electromyogram (EMG) (bilateral submental muscles) signals at 256 Hz. Sleep analysis was performed with RemLogic-E™ software, following AASM Version 2.0 criteria (Berry et al., 2012). Data were segmented into 30-s epochs, with signals filtered at 0.3–35 Hz (EEG), 10–100 Hz (EOG, EMG), and a 50 Hz notch filter applied. CI criteria followed ICSD-3 standards (SOL >30 min and/or TA >30 min) (Sateia, 2014). Sleep quality measures included: SOL (time from “start” to sleep onset), TST (total sleep time, including 4–6 cycles), LST (time in stages 1 and 2 NREM), DST (time in stages 3 and 4 NREM), REM (time in REM), AT (time awake after sleep onset), and SE (sleep efficiency, ratio of actual sleep time to time in bed) (Berry et al., 2012).

#### 2.4.3 HRV measurement and analysis

Heart rate variability is commonly used to assess the balance between sympathetic and parasympathetic nervous activity (Santos-De-Araújo et al., 2022). In accordance with established guidelines, HRV was measured at the same time of day before sleep for a period of 5 min in a quiet room, whilst participants were awake, lying supine and breathing spontaneously (Laborde et al., 2017). R–R intervals (the time between R waves in two QRS complexes) were recorded for no less than 10 min using a Polar H10 heart rate chest strap (Polar Electro, Finland). The data were processed and analyzed using the Kubios HRV-standard software, extracting 5-min segments to calculate HRV and assess autonomic nervous status. This included measures of Mean R-R (MRR), Root Mean Square of Successive RR Interval Differences (RMSSD), SDNN (Standard Deviation of NN Intervals), TINN (Triangular Interpolation of NN Interval Histogram), PNN50% (Percentage of Successive NN Intervals that Differ by More than 50 m), High Frequency (HF), Mean Heart Rate (MHR), Stress Index (SI), Low Frequency (LF), LF/HF Ratio (Ratio between sympathetic and parasympathetic nervous system activity). Approximate Entropy (ApEn) and Sample Entropy (SampEn): Non-linear measures that evaluate the complexity and irregularity of HRV patterns.

#### 2.4.4 HAMA and HAMD scales

The HAMA (Thompson, 2015) and the HAMD (Zimmerman et al., 2013) scales are commonly used clinical instruments for assessing levels of anxiety and depression. In this study, these scales were used to exclude participants with sleep-related complications caused by anxiety or depressive symptoms. The HAMA consists of 14 items, and the HAMD consists of 17 items. Both scales use a five-point Likert scoring system (0–4), corresponding to “no symptoms,” “mild symptoms,” “moderate symptoms,” “marked symptoms,” and “severe symptoms,” respectively.

### 2.5 Model development

Binary logistic regression model: CI was initially diagnosed based on the ICSD-3 criteria, alongside PSG data. CI status (1 = CI present, 0 = no CI) was treated as the dependent variable, and pre-sleep HRV variables, including HF, LF, LF/HF Ratio; Time-domain: MRR, RMSSD, SDNN, TINN, PNN50%, MHR, SI; Non-linear: ApEn and SampEn were used as independent variables for binary logistic regression analysis. Data were randomly divided into a training set (80%) and a validation set (20%) to perform a train-validation split. The stepwise method is employed for variable selection. In the model formula, p represents the probability of CI (0/1 = 1), and 1−p represents the probability of no CI (0/1 = 0). The Hosmer-Lemeshow (HL) test was used to evaluate the model’s goodness-of-fit. *R* ^2^ values indicate the fit: The goodness-of-fit of the binary logistic regression model was assessed through using the *R* ^2^ value. Potential multicollinearity among the independent variables was assessed using variance inflation factors (VIF), and variables with VIF values exceeding the threshold of 10 were excluded from the analysis to ensure the robustness of the model. To evaluate how well independent variables explained the dependent variable, model fit was classified using *R* ^2^ as follows: poor fit (<0.2), low fit (0.2–0.5), moderate fit (0.5–0.8) and high level of fit (>0.8).

Multiple linear regression model: Data were first normalized (X-Min)/(Max-Min). HRV measures were set as independent variables, and measures of sleep quality were treated as dependent variables. Data were randomly divided into a training set (80%) and a validation set (20%) to perform a train-validation split. The *R* ^2^ value ranges from 0 to 1, with higher values indicating better fit. The tolerance of the prediction equation must exceed 0.1, and the VIF must be less than 10, indicating no multicollinearity. The Durbin-Watson test values ranged from 0 to 4, demonstrating independence between variables and the absence of autocorrelation.

Neural network model: The Multi-Layer Perceptron (MLP) neural network model was constructed by randomly dividing data into a training set (80%) and a validation set (20%) to perform a train-validation split. The training data were normalized (X-Min)/(Max-Min) to ensure uniform data scaling. The activation function was ReLU, and weights were optimized using the Adam optimizer with an initial learning rate of 0.001. L2 regularization was set at 0.0001, the maximum number of iterations was 200, and the optimization tolerance was 0.0001. The current model architecture employed a single hidden layer comprising 100 neurons, which is a similar approach to earlier studies involving comparable data structures and sample sizes (Sheela and Deepa, 2013). To evaluate the robustness of this configuration, we also tested alternative architectures with varying numbers of hidden neurons (e.g., 50, 150, and 200) as well as models with multiple hidden layers. However, these modifications did not further enhance performance.

#### 2.5.1 Model construction and validation

A train-validation split of the binary logistic regression, multiple linear regression, and neural network models were carried out by firstly randomly dividing data into a training set (80%) and validation set (20%). The validation methodology for both the MLP neural network and multiple linear regression models was executed as follows: The training data set was first used to construct the models, and the validation data set (HRV) was then incorporated into the formula obtained from the training set to generate the predicted values. Parameters of sleep quality measured by the PSG validation set were tested for consistency by comparing them to the sleep parameters predicted by the validation data set. The performance of multiple linear regression and MLP neural network models was evaluated and compared using the following metrics: Mean Absolute Error (MAE), Root Mean Squared Error (RMSE) and Mean Absolute Percentage Error (MAPE). Using the reserved 20% validation subset, the binary logistic regression model (fitted on the training set) was validated by applying its coefficients to the HRV data to compute event probabilities. These probabilities were converted to binary classifications using a decision threshold of 1 (1 = CI present, 0 = no CI). Model discrimination was assessed via the Area Under the Receiver Operating Characteristic Curve (AUC-ROC).

### 2.6 Statistical analysis

Data normality was first assessed using the Kolmogorov-Smirnov test. Pearson correlation coefficients were used to evaluate the relationship between observed and predicted sleep quality. The magnitudes of the correlations were classified as follows: trivial (<0.1), small (0.1–0.3), moderate (0.3–0.5), large (0.5–0.7), very large (0.7–0.9), and extremely large (>0.9) (Hopkins et al., 2009). Bland-Altman analyses were used to assess reliability. The mean and difference between observed and predicted sleep quality values were then calculated to establish the limits of agreement (95% distribution range), and 95% limits of agreement (95% LoA) were constructed (Giavarina, 2015). Paired t-tests were used to compare the adaptation of PSQI, while independent t-tests were used to compare the differences in HRV and sleep quality (PSG) between groups. Data are reported as mean ± standard deviation (M ± SD). The skew distribution is expressed as the median (upper quartile to lower quartile). Statistical significance was accepted as α < 0.05. All statistical analyses were performed using SPSS 26.0.

## 3 Results

### 3.1 Sleep and pre-sleep HRV characteristics

The data of 12 participants were excluded due to electrode detachment and erroneous PSG data, and nine did not complete the experiment. There were no significant differences between CI and normal sleep groups in age, height, or weight (*P* > 0.05). The absence of significant differences in PSQI scores between the screening phase and the month prior to study participation indicated that there were no differences in sleep disturbances and sleep quality prior to commencing the study. The PSQI scores of the CI group were significantly higher than those of the normal sleep group (CI group: 10.96 ± 2.13 vs. normal sleep group: 4.04 ± 1.69*, P* < 0.01), with our PSG data indicating that the prevalence of CI among male athletes in this study was 56% (95% CI: 49%–64%). When compared to the normal sleep group, the CI group showed an increased duration of SOL (*P* < 0.001, mean difference = 11.94 min, *d* = 0.68), and TA (*P* < 0.001, mean difference = 41.68 min, *d* = 2.28). In addition, there was also a decrease in the duration of TST (*P* < 0.001, mean difference = −42.94 min, *d* = 0.84), SE (*P* < 0.001, mean difference = −10.4%, *d* = 2.18), and DST (*P* < 0.001, mean difference = −3.7%, *d* = 1.133). Athletes with CI also exhibited lower values for a range of pre-sleep HRV measures when compared to the normal sleep group, including MRR (*P* < 0.001, mean difference = −213.68 m, *d* = 1.68), RMSSD (*P* < 0.001, mean difference = −49.35 m, *d* = 2.01), PNN50 (*P* < 0.001, mean difference = −41.84%, *d* = 2.86), ApEn (*P* < 0.001, mean difference = −0.23, *d* = 1.66), and SampEn (*P* < 0.001, mean difference = −0.35, *d* = 1.49) (Table 1).

**TABLE 1:** Sleep, HRV and EEG test results of athletes included in the model (M ± SD).

Variable type	Dependent variable	Group summary	Normal sleep	CI	P Value	Cohen’s d
Sleep quality	TST/min	421.54 ± 55.51	445.72 ± 44.69	402.78 ± 56.02***	<0.001	0.835
	SOL/min	20.91 ± 18.60	14.19 ± 9.50	26.13 ± 22.01***	<0.001	0.675
	TA/min	42.67 ± 27.61	19.20 ± 7.04	60.87 ± 23.55***	<0.001	2.279
	SE/%	89.55 ± 7.04	95.42 ± 1.76	85.00 ± 6.17***	<0.001	2.182
	LST/%	61.32 ± 6.36	62.75 ± 5.76	60.21 ± 6.61	0.008	0.407
	DST/%	22.34 ± 3.71	24.41 ± 3.22	20.74 ± 3.26***	<0.001	1.133
	REM/%	16.34 ± 3.89	15.67 ± 3.80	16.86 ± 3.90	0.045	0.308
HRV	MRR/ms	901.02 ± 165.42	1,021.37 ± 125.19	807.69 ± 128.60***	<0.001	1.681
	SDNN/ms	46.14 ± 26.54	43.65 ± 23.36	48.04 ± 28.71	0.282	0.165
	RMSSD/ms	52.24 ± 34.69	80.04 ± 30.36	30.68 ± 18.94***	<0.001	2.008
	PNN50/%	26.36 ± 25.41	49.93 ± 19.19	8.09 ± 9.73***	<0.001	2.860
	TINN/ms	235.27 ± 136.74	234.22 ± 150.16	236.08 ± 126.13	0.931	0.014
	HF/Hz	0.26 ± 0.07	0.26 ± 0.07	0.26 ± 0.06	0.723	0.056
	MHR/bpm	68.89 ± 12.67	69.59 ± 13.29	68.35 ± 12.20	0.522	0.098
	SI	12.02 ± 7.98	12.61 ± 8.66	11.55 ± 7.42	0.388	0.132
	LF/Hz	0.09 ± 0.08	0.09 ± 0.12	0.08 ± 0.02	0.248	0.177
	LF/HF	1.96 ± 2.67	2.17 ± 3.27	1.80 ± 2.10	0.371	0.137
	ApEn	1.03 ± 0.18	1.16 ± 0.09	0.93 ± 0.17***	<0.001	1.663
	SampEn	1.70 ± 0.29	1.89 ± 0.17	1.54 ± 0.27***	<0.001	1.485

Significant differences between CI, and normal sleep groups are denoted as follows: *P < 0.05,**P < 0.01,**P < 0.001. Abbreviations: total sleep time, TST; sleep onset latency, SOL; time awake, TA; sleep efficiency, SE; light sleep time, LST; deep sleep time, DST; rapid eye movement, REM; mean R-R, MRR; standard deviation of NN, SDNN; root mean square of successive rr interval differences, RMSSD; percentage of successive nn intervals that differ by more than 50 m, PNN50; triangular interpolation of nn interval histogram, TINN; high frequency, HF; mean heart rate, MHR; stress Index, SI; low frequency, LF; approximate entropy, ApEn; sample entropy, SampEn.

### 3.2 Binary logistic regression model for the prediction of CI

Binary logistic regression analysis was conducted using measures of HRV as independent variables and the presence of CI (0/1) as the dependent variable (Table 2). The model automatically filters out RMSSD, PNN50, MHR, and SampEn, as they are all negatively correlated with CI (*R* ^2^ = 0.902). The HL goodness-of-fit test indicated a good model fit (*P* = 1.000 > 0.05). The final model equation is as follows: ln (p/1-p) = 28.317–0.073*RMSSD-0.222*PNN50-11.436*SampEn. The accuracy of using HRV measures to predict CI was 96%, respectively (Table 3). Using the validation set, the integrated diagnostic model achieved an accuracy of 97.14% and an excellent discriminative ability (AUC = 0.997). At the optimal threshold (0.493), both sensitivity and specificity exceeded 96%, indicating strong clinical applicability. In contrast, individual HRV metrics (RMSSD, PNN50, and SampEn) performed at chance level, with AUCs ranging from 0.023 to 0.072, highlighting their limited diagnostic value when used in isolation.

**TABLE 2:** Binary logistic regression results of HRV before sleep for predicting CI.

Independent variable	Regression coefficient	Standard error	z-value	Wald	p-value	Odds ratio	95% CI for OR	VIF	Tolerance
RMSSD/ms	−0.073	0.028	−2.572	6.615	0.010	0.930	0.880–0.983	1.615	0.619
PNN50/%	−0.222	0.084	−2.657	7.057	0.008	0.801	0.680–0.943	1.638	0.610
SampEn	−11.436	4.737	−2.414	5.828	0.016	0.000	0.000–0.116	1.459	0.685

McFadden *R* ^2^ = 0.902. Abbreviations: root mean square of successive rr interval differences, RMSSD; percentage of successive nn intervals that differ by more than 50 m, PNN50; mean heart rate, MHR; sample entropy, SampEn.

**TABLE 3:** Accuracy of the binary logistic regression model for predicting insomnia based on pre-sleep HRV.

Numeric type		Predicted value		Prediction accuracy	Prediction error rate
		0	1		
Actual Value	0	56	3	94.91%	5.09%
	1	3	77	96.25%	3.75%
Summary				95.58%	4.42%

### 3.3 Multiple linear regression model for predicting sleep quality

A multiple linear regression model was developed using pre-sleep HRV measures as independent variables and sleep quality as the dependent variable (Table 4). All model variables satisfied the conditions of variable importance in projection (VIP <10), tolerance >0.1, and D-W statistics within the range of 0–4, indicating no multicollinearity issues. The model demonstrated a moderate fit for predicting TA (*R* ^2^ = 0.526, *P* < 0.001) and SE (*R* ^2^ = 0.481, *P* < 0.001). The remaining measures of TST, SOL, LST, DST and REM exhibited poor predictive values, with *R* ^2^ values below 0.2.

**TABLE 4:** Multiple linear Regression Model of Pre-sleep HRV for Predicting Sleep Quality.

Dependent variable	Regression equation	Adjusted *R* ^2^	F	p-value	VIF	Tolerance	D-W	Standardized β	95% CI
TST	= 0.422 + 0.390* RMSSD	0.109	16.768	<0.001	1	1	1.772	0.330	0.203–0.576
SOL	= 0.243–0.270* RMSSD	0.093	14.034	<0.001	1	1	1.199	−0.305	−0.412 ∼ −0.129
TA	= 0.892–0.588* PNN50-0.300* MHR-0.267*ApEn	0.526	49.849	<0.001	1.006–1.453	0.688–0.995	1.794	PNN50: 0.577 MHR: 0.193 ApEn: 0.201	−0.730 ∼ −0.446 −0.482 ∼ −0.119 −0.453 ∼ −0.081
SE	= 0.502 + 0.235* RMSSD+0.332* PNN50+0.201* MHR	0.481	41.678	<0.001	1.010–1.498	0.668–0.990	1.616	RMSSD:0.204 PNN50: 0.515 MHR: 0.204	0.085–0.385 0.237–0.428 0.080–0.322
LST	= 0.205 + 0.340* SampEn	0.074	10.992	0.001	1	1	1.661	0.273	0.139–0.542
DST	= 0.374 + 0.201* MRR+0.194*ApEn	0.16	12.912	<0.001	1.347	0.742	1.593	MRR: 0.215 ApEn: 0.245	0.034–0.369 0.052–0.336
REM	= 0.708–0.384* SampEn	0.069	10.185	0.002	1	1	1.567	−0.263	−0.621 ∼ −0.148

Abbreviations: total sleep time, TST; sleep onset latency, SOL; time awake, TA; sleep efficiency, SE; light sleep time, LST; deep sleep time, DST; rapid eye movement, REM; mean R-R, MRR; root mean square of successive rr interval differences, RMSSD; percentage of successive nn intervals that differ by more than 50 m, PNN50; high frequency, HF; mean heart rate, MHR; approximate entropy, ApEn; sample entropy, SampEn.

**FIGURE 1:**
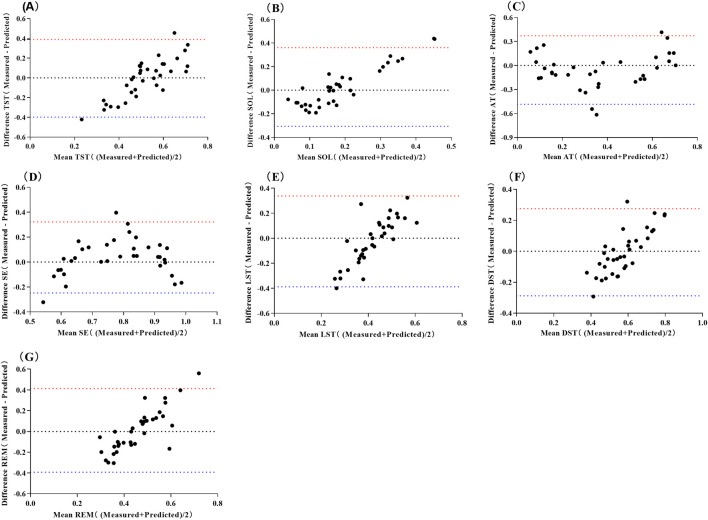
Bland-Altman Scatter Plot for Predicting Sleep Structure Using Multiple Linear Regression Models of Pre-sleep HRV. Abbreviations: **(A)** total sleep time, TST; **(B)** sleep onset latency, SOL; **(C)** time awake, TA; **(D)** sleep efficiency, SE; **(E)** light sleep time, LST; **(F)** deep sleep time, DST; **(G)** rapid eye movement, REM. The red line represents the upper limit of the 95% LoA (1.96 SD), while the blue line represents the lower limit of the 95% LoA (1.96 SD).

The predictive equation was validated by applying HRV data from a validation set of 35 cases (20%) (Table 5). The actual and predicted values of SOL demonstrated a moderate positive correlation (*P* < 0.01), while the actual and predicted values of TA, SE, and DST showed a strong positive correlation (*P* < 0.01). No correlations were observed between the predicted and actual values for LST and REM (*P* > 0.05). The Bland-Altman scatterplot (Figure 1) revealed that the probability of agreement within the limits of consistency for all predicted values ranged from 91%–97%.

**TABLE 5:** Multiple linear Regression Results of Pre-sleep HRV for Predicting Sleep Quality.

Dependent variable	The boundary probability for consistency									
	Actual value	Predicted value	MAE	RMSE	MAPE	r	Lower limit	Upper limit	Probability within consistency limits	bias
TST	0.52 ± 0.21	0.52 ± 0.07	0.101	0.120	0.155	0.248	−0.398	0.390	94.29%	−0.004
SOL	0.20 ± 0.19	0.18 ± 0.05	0.127	0.153	0.287	0.459**	−0.306	0.361	94.29%	0.028
TA	0.34 ± 0.26	0.39 ± 0.22	0.207	0.254	0.516	0.554***	−0.486	0.372	91.43%	−0.057
SE	0.80 ± 0.16	0.76 ± 0.14	0.083	0.104	0.109	0.536***	−0.248	0.322	94.29%	0.037
LST	0.41 ± 0.17	0.43 ± 0.05	0.142	0.174	0.337	−0.101	−0.388	0.338	97.14%	−0.025
DST	0.57 ± 0.17	0.58 ± 0.07	0.136	0.163	0.284	0.602***	−0.288	0.276	94.29%	−0.006
REM	0.46 ± 0.20	0.45 ± 0.06	0.13	0.159	0.313	0.080	−0.392	0.412	97.14%	0.010

Abbreviations: total sleep time, TST; sleep onset latency, SOL; time awake, TA; sleep efficiency, SE; light sleep time, LST; deep sleep time, DST; rapid eye movement, REM; mean absolute error, MAE; root mean squared error, RMSE; mean absolute percentage error, MAPE.

### 3.4 Multiple linear regression model for predicting sleep quality

**FIGURE 2:**
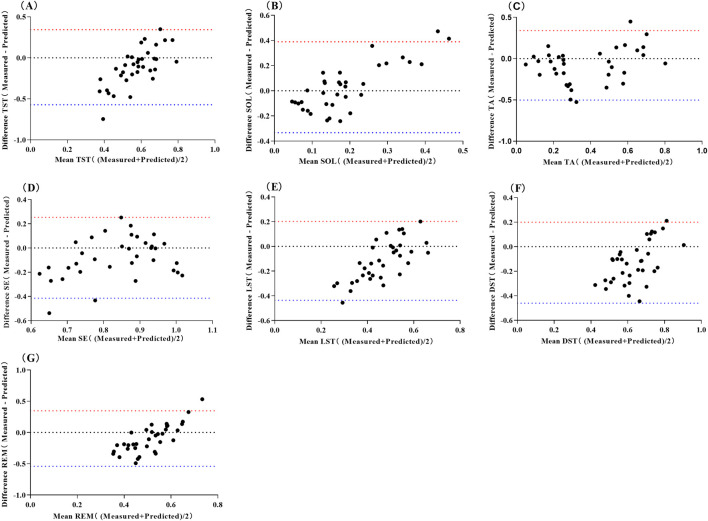
Bland-Altman Scatter Plot for Predicting Sleep Quality Using Neural Network Model of Pre-sleep HRV Abbreviations: **(A)** total sleep time, TST; **(B)** sleep onset latency, SOL; **(C)** time awake, TA; **(D)** sleep efficiency, SE; **(E)** light sleep time, LST; **(F)** deep sleep time, DST; **(G)** rapid eye movement, REM. The red line represents the upper limit of the 95% LoA (1.96 SD), while the blue line represents the lower limit of the 95% LoA (1.96 SD).

The pre-sleep TA prediction values had a strong positive correlation with the measured values (*P* < 0.01), while the prediction values for other sleep parameters showed no significant correlation with their measured values (*P* > 0.05) (Table 6). The mean absolute error range between the measured and predicted values was 0.120–0.269, the root mean square error range was 0.157–0.299, the mean absolute percentage error range was 0–1.059, and the bias range was −0.174 to 0.028. The results of the consistency test indicated that the boundary probability ranges were between 94% and 97% (Figure 2).

**TABLE 6:** Neural network model of pre-sleep HRV for predicting sleep quality.

Dependent variable	The boundary probability for consistency									
	Actual value	Predicted value	r	MAE	RMSE	MAPE	Lower Limit	Upper Limit	Probability within consistency limits	bias
TST	0.52 ± 0.21	0.63 ± 0.08	−0.03	0.188	0.255	0.136	−0.571	0.343	94.29%	−0.114
SOL	0.20 ± 0.19	0.17 ± 0.07	0.19	0.142	0.176	0.152	−0.333	0.390	94.29%	0.028
TA	0.34 ± 0.26	0.42 ± 0.20	0.555***	0.170	0.220	0.182	−0.502	0.341	94.29%	−0.080
SE	0.80 ± 0.16	0.88 ± 0.12	0.319	0.142	0.184	0.077	−0.415	0.253	94.29%	−0.081
LST	0.41 ± 0.17	0.52 ± 0.07	0.315	0.161	0.189	0.221	−0.436	0.202	97.14%	−0.117
DST	0.57 ± 0.17	0.70 ± 0.09	0.311	0.186	0.219	0.145	−0.460	0.200	97.14%	−0.130
REM	0.46 ± 0.20	0.56 ± 0.07	−0.16	0.188	0.235	0.201	−0.540	0.347	97.14%	−0.097

Caption -Abbreviations: total sleep time, TST; sleep onset latency, SOL; time awake, TA; sleep efficiency, SE; light sleep time, LST; deep sleep time, DST; rapid eye movement, REM., mean absolute error, MAE; root mean squared error, RMSE; mean absolute percentage error, MAPE.

## 4 Discussion

This study aimed to investigate whether pre-sleep HRV could predict CI and a range of sleep quality indicators in male national level team-sport athletes. We hypothesized that pre-sleep HRV would reliably predict both CI and sleep quality outcomes. Consistent with our hypothesis, the analysis using pre-sleep HRV measures within a binary logistic regression model demonstrated an accurate prediction of CI. Furthermore, stepwise multiple linear regression moderately predicted sleep continuity measures of time awake and SE, but was poor at predicting sleep stages, such as LST, DST and REM. In general, the stepwise multiple linear regression outperformed the current neural network model at predicting measures of sleep quality, but the neural network model was superior at predicting TA. Taken together, these findings demonstrate that pre-sleep HRV accurately predicts CI and sleep continuity in male national level athletes.

The present study suggests that the prevalence of CI among Chinese national level athletes is between 49% and 64%, which is substantially higher than the rates reported in previous studies using elite athletes (5%–33%) (Gerber et al., 2022; Gupta et al., 2017). Such large discrepancies are likely due to PSG being more accurate than subjective measures for detecting CI (Sateia, 2014). For example, our earlier research, which utilized sleep-monitoring mattresses, revealed that the incidence of sleep disorders among Chinese elite bobsleigh athletes was ∼63% (Li et al., 2022). As expected, CI was accompanied by poor sleep quality in the form of reductions in TST, SE, and DST and increases in SOL and TA (Table 1). These observations are particularly important considering that CI is prevalent among athletes and has been linked with impairments in physical performance, attention, concentration and memory (Leger et al., 2005). The present study also observed that pre-sleep HRV parameters, RMSSD, PNN50, ApEn, and SampEn were reduced in athletes suffering from CI (Table 1). This suggests an imbalance in autonomic nervous system regulation, specifically a downregulation of parasympathetic and an upregulation of sympathetic activity. Since autonomic dysfunction is known to contribute to overreaching (Achten and Jeukendrup, 2003), these findings imply that CI may increase an athlete’s vulnerability to overtraining which can impair athletic performance and increase the risk of injury and illness (Soligard et al., 2016).

To our knowledge, this is the first study to demonstrate that pre-sleep HRV can predict CI in national level athletes using a binary logistic regression model. This is of importance as predicting CI across a range of different team-sports is challenging due to the complex interplay of individual-level factors, including sport type, performance level, training workload, sleep habits and personal beliefs (Walsh et al., 2021). Binary logistic regression models have been shown to predict CI in response to cognitive behavioral therapy with accuracies ranging from 60%–71% when used with the general public (Holler et al., 2024). In contrast, we showed a considerably higher accuracy of 96% through using pre-sleep HRV. However, caution is advised when interpreting these findings given the high predictive performance for CI as it may partly reflect hidden overlap between the training and validation datasets. Although we used separate data sets, these data were drawn from similar athlete populations (e.g., age, sex, team-based sports and national level) under similar measurement conditions, therefore the accuracy of our model could have been inflated. This being said, the higher predictive accuracy of pre-sleep HRV may also reflect that parasympathetic and sympathetic systems responding more clearly to stress, fatigue, or psychological strain in national-level athletes, making HRV a more sensitive marker of CI in this population. In this regard, our previous research showed that national level athletes frequently exhibit negative emotional states (Li et al., 2022), and there is well-established link between negative emotions and HRV (Di Simplicio et al., 2012).

The current study also demonstrated that multiple linear regression could predict parameters of TA and SE, which are key indicators of sleep continuity. These results align to the small body of research that has investigated whether pre-sleep HRV is related to sleep quality in healthy young and middle-aged adults (Jung et al., 2017; Fantozzi et al., 2019; Werner et al., 2015). For instance, Fantozzi et al. (2019) found that measures of parasympathetic activity, such as RMSSD and PNN50 predicted a shorter wake after sleep onset time. Similarly, in the present study, PNN50 was an integral component of the multiple linear regression model to predict both TA and SE, in addition to RMSSD for the prediction of SE. We expanded on previous work by identifying additional pre-sleep HRV predictors for sleep continuity (TA: PNN50, MHR, ApEn; SE: PNN50, MHR, RMSSD). However, pre-sleep HRV was less effective in predicting sleep phases (TST, LST, and REM). Nevertheless, the multiple linear regression model outperformed our MLP neural network model for predicting SOL, SE, and DST. This suggests that a linear relationship exists between pre-sleep HRV and some sleep related outcomes. Although the MLP neural network model was superior at predicting TA, which could also indicate that this type of model is better at solving more complex nonlinear problems. Whilst these results are promising, collectively they indicate that a single physiological signal, such as HRV, may restrict the predictive potential of a model when assessing measures of sleep quality which are multidimensional and complex in nature.

## 5 Limitations and future directions

While this study offers important insights into sleep prediction among athletes, several limitations should be considered. All regression models are inherently susceptible to overfitting, in which the model captures spurious patterns in the training data, thereby reducing its generalizability to independent datasets. As our work was considered exploratory, we performed a train-validation split to efficiently provide an estimate of model performance on unseen data, helping to detect overfitting. However, future work should use cross-validation to evaluate the models across multiple splits, providing a more stable and accurate estimate of their performance. The sample of participants in the current study was limited to male Chinese athletes involved in team sports, which may influence the broader applicability of our findings to other athletic or non-athletic populations, including female athletes or those in individual sports. Additionally, the study utilized HRV as the sole physiological indicator, without accounting for other psychophysiological (e.g., circadian rhythm, blood pressure, stress and anxiety), which could play a key role in sleep regulation. To strengthen the validity and generalizability of future models, research should include more diverse cohorts and a wider range of physiological and psychological variables. Incorporating alternative sleep assessment tools, such as actigraphy and subjective measures like sleep diaries, may also enhance ecological validity and provide a more holistic understanding of sleep in athletic populations.

## 6 Conclusion

The current binary logistic regression model accurately predicted CI in male national level athletes. In addition, our multiple linear regression model was effective at predicting sleep continuity measures (TA and SE), but was poor at predicting sleep phases (TST, LST, DST and REM). Based on these findings, pre-sleep HRV could be a potential approach for practitioners and coaches to accurately predict CI, enabling the timely implementation of strategies to enhance sleep quality and, consequently, maintain optimal athletic performance.

## Data Availability

The raw data supporting the conclusions of this article will be made available by the authors, without undue reservation.
